# Evolving thermostability in mutant libraries of ligninolytic oxidoreductases expressed in yeast

**DOI:** 10.1186/1475-2859-9-17

**Published:** 2010-03-18

**Authors:** Eva García-Ruiz, Diana Maté, Antonio Ballesteros, Angel T Martinez, Miguel Alcalde

**Affiliations:** 1 Department of Biocatalysis, Institute of Catalysis, CSIC, 28049 Madrid, Spain; 2 Centro de Investigaciones Biológicas, CSIC, 28040 Madrid, Spain

## Abstract

**Background:**

In the picture of a laboratory evolution experiment, to improve the thermostability whilst maintaining the activity requires of suitable procedures to generate diversity in combination with robust high-throughput protocols. The current work describes how to achieve this goal by engineering ligninolytic oxidoreductases (a high-redox potential laccase -HRPL- and a versatile peroxidase, -VP-) functionally expressed in *Saccharomyces cerevisiae*.

**Results:**

Taking advantage of the eukaryotic machinery, complex mutant libraries were constructed by different *in vivo *recombination approaches and explored for improved stabilities and activities. A reliable high-throughput assay based on the analysis of T_50 _was employed for discovering thermostable oxidases from mutant libraries in yeast. Both VP and HRPL libraries contained variants with shifts in the T_50 _values. Stabilizing mutations were found at the surface of the protein establishing new interactions with the surrounding residues.

**Conclusions:**

The existing tradeoff between activity and stability determined from many point mutations discovered by directed evolution and other protein engineering means can be circumvented combining different tools of *in vitro *evolution.

## Background

During the last couple of decades, thermostability has been considered by many as a key feature in terms of protein robustness, evolvability and catalytic function [1-4]. From a practical point of view, the engineering of thermo-tolerant biocatalysts is highly desirable since transformations at high temperatures intrinsically supply a box-set of key biotechnological advantages (higher entropies -better reaction yields-, solubilisation of hydrophobic compounds or low levels of microbial side-contamination, among others). Besides, thermostable enzymes are typically tolerant to many other harsh conditions often required in industry, such as the presence of organic co-solvents, extreme pHs, high salt concentrations, high pressures, etc [5,6]. Few exceptions aside [7,8], the discovery of stabilizing mutations is not always straightforwardly accomplished without significant drops in turnover rates [9]. Most of these mutations, which establish new interactions by salt bridges, hydrogen bonds, hydrophobic contacts or even disulfide bridges, are placed either at the protein surface or in internal cores pursuing the tightly packing of the tertiary protein structure in order to prevent unfolding and denaturation under extreme environments [10]. On the contrary, improvements in activity are generally accomplished by introducing beneficial but destabilizing mutations in *hot *regions for catalysis (substrate binding sites, channels of access to the active pockets) although sometimes distant mutations can also vary the catalytic function by altering the dynamics and geometry in the protein scaffold [3]. There are several examples in literature about the stabilization of enzymes by directed evolution or rational design but unfortunately, main constraints still remain from the lack of appropriate methods to recreate diversity in conjunction with reliable screening strategies, especially if one wants to surpass the existing tradeoff between activity and thermostability for many single residue substitutions [10-20].

Among the enzymes forming the ligninolytic system of white-rot fungi (*i.e*. involved in lignin biodegradation), high redox potential laccases HRPL (EC 1.10.3.2) and peroxidases, including versatile peroxidases (VP; EC 1.11.1.14) are outstanding biocatalysts finding potential applications in paper pulp bleaching and functionalization, bioremediation, organic synthesis, food and textile industries, nanobiodevice construction and more [21-23]. Indeed, HRPL can oxidize dozens of different compounds releasing water as the only by-product and in the presence of redox mediators (diffusible electron carriers from natural or synthetic sources) their substrates specificities are further expanded [24,25]. On the other hand, VP (with redox potential above +1000 mV) shares the catalytic features of lignin and manganese peroxidase in terms of substrate specificity, together with the ability to oxidize phenols and dyes characteristic of low redox-potential peroxidases. Indeed, the presence of different catalytic sites in a small and compact protein structure (around 300 amino acids) makes VP an ideal platform for laboratory evolution strategies [23,26,27].

Here, we have employed these two enzymatic systems as departure points to improve their protein thermostability by directed evolution. VP and HRPL were functionally expressed in yeast and mutant libraries were constructed combining several methodologies of *in vitro *evolution to guarantee the library complexity, favoring the selection of optimal crossover events or the discovery of beneficial mutations. Highly functional/soluble expressed mutants were stressed under high temperatures and explored for activity and stability. The analysis of the data from screening (ratio residual activity/initial activity in combination with the T_50 _values) enabled us to discover stabilizing mutations in both systems.

## Materials and methods

HRPL from basidiomycete PM1 [28] (PM1-7H2 mutant) and VP from *Pleurotus eryngii *(10C3, 6B1, 13E4, 6E7 and 11F3 mutants of the allelic variant VPL2, GenBank AF007222) were used as parent types for library construction. Both systems are from previous engineering work by several rounds of directed evolution in *S. cerevisiae *including the replacement of their original native signal sequences by the alpha factor prepro-leader, ([29] and unpublished material). ABTS (2,2'-azino-bis(3-ethylbenzothiazoline-6-sulfonic acid)), bovine haemoglobine, Taq polymerase and the *S. cerevisiae *transformation kit were purchased from Sigma-Aldrich (Madrid, Spain). The *E. coli *XL2-blue competent cells and the Genemorph Random mutagenesis kit were from Stratagene (La Jolla, CA, USA). The protease deficient *S. cerevisiae *strain BJ5465 was from LGCPromochem (Barcelona, Spain). The uracil independent and ampicillin resistance shuttle vector pJRoC30 was obtained from the California Institute of Technology (CALTECH, USA), while the zymoprep yeast plasmid miniprep kit, zymoclean gel DNA recovery kit, and the DNA clean and concentrator TM-5 kit were all from Zymo Research (Orange, CA). NucleoSpin Plasmid kit was purchased from Macherey-Nagel (Germany) and the restriction enzymes *BamH*I and *Xho*I were from New England Biolabs (Hertfordshire, UK). All chemicals were of reagent-grade purity.

### 1. Culture media

Minimal medium contained 100 mL 6.7% sterile yeast nitrogen base, 100 mL 19.2 g/L sterile yeast synthetic drop-out medium supplement without uracil, 100 mL sterile 20% raffinose, 700 mL *sdd*H_2_O and 1 mL 25 g/L chloramphenicol. YP medium contained 10 g yeast extract, 20 g peptone and *dd*H_2_O to 650 mL. Expression medium contained 720 mL YP, 67 ml 1 M KH_2_PO_4 _pH 6.0 buffer, 111 mL 20% galactose, 1 ml 25 g/L chloramphenicol and *dd*H_2_O to 1000 mL. For HRPL the expression medium was supplemented with 2 mM CuSO_4 _and 25 g/L ethanol. For VP the expression medium was supplemented with 100 mg/L bovine haemoglobine. YPD solution contained 10 g yeast extract, 20 g peptone, 100 mL 20% sterile glucose, 1 ml 25 g/L chloramphenicol and *dd*H_2_O to 1000 mL. SC drop-out plates contained 100 mL 6.7% sterile yeast nitrogen base, 100 mL 19.2 g/L sterile yeast synthetic drop-out medium supplement without uracil, 20 g bacto agar, 100 mL 20% sterile glucose, 1 mL 25 g/L chloramphenicol and *dd*H_2_O to 1000 ml.

### 2. Library construction for laboratory evolution

#### General aspects

Unless otherwise specified, PCR fragments were cleaned, concentrated and loaded onto a low melting point preparative agarose gel and purified using the Zymoclean gel DNA recovery kit (Zymo Research). PCR products were cloned under the control of the Gal 10 promoter of the expression shuttle vector pJRoC30, replacing the corresponding parental gene in pJRoC30. To remove the parental gene, the pJRoC30 plasmid was linearized (with *Xho*I and *BamH*I for HRPL- and VP-libraries). Linearized vector was concentrated and purified as described above for the PCR fragments.

Mutagenic *StEP *(*Staggered Extension Process*) followed by *in vivo *DNA shuffling and *IvAM *(*In vivo A*ssembly of *M*utant libraries with different mutational spectra) were used to create the VP and HRPL libraries, respectively, as described below.

#### VP Library: mutagenic *StEP *+ *in vivo *DNA shuffling

10C3, 6B1, 13E4, 6E7 and 11F3 VP-mutants were used as parental types. *StEP *was performed as reported elsewhere [30] with some modifications. In order to favor random mutagenesis during *StEP*, Taq DNA-polymerase was employed for the PCR reaction along with low concentration of templates to promote the introduction of point mutations during the amplification. The primers used were: RMLN-sense (5'-CCTCTAATACTTTAACGTCAAGG-3') and RMLC-antisense (5'-GGGAGGGCGTGAATGTAAGC-3'). For the *in vivo *ligation, overhangs of 40 bp and 66 bp that were homologous to linearized vector were designed. PCR reactions were performed in a final volume of 50 μL containing 90 nM RMLN, 90 nM RMLC, 0.3 mM dNTPs, 3% dimethylsulfoxide (DMSO), 0.05 U/μL of Taq polymerase (Sigma), 1.5 mM MgCl_2 _and 0.1 ng/μL of 10C3, 6B1, 13E4, 6E7 and 11F3 DNA-template mixture. *StEP *was carried out using a gradient thermocycler (Mycycler, Biorad, USA). The thermal cycling parameters were as follows: 95°C for 5 min (1 cycle), 94°C for 30 s and 55°C for 20 s (90 cycles). Purified PCR products were further recombined by *in vivo *DNA-shufflling [31]. PCR mutated/recombined products were mixed equimolarly (160 ng of each product) and transformed along with linearized vector (ratio PCR product:vector, 4:1) into competent cells using the yeast transformation kit (Sigma). A mutant library of ~2000 clones was explored.

#### HRPL Library: *IvAM*

*IvAM *(~1300 clones) was performed as reported elsewhere [32] with some modifications. HRPL PM1-7H2 mutant was used as parent type. Mutagenic PCR was carried out using the following thermal cycling parameters: 95°C for 2 min (1 cycle), 94°C for 0.45 min, 53°C for 0.45 min, 74°C for 3 min (28 cycles), 74°C for 10 min (1 cycle). For the Taq library the concentrations of each ingredient in 50 μL final volume were as follows: 90 nM RMLN; 90 nM RMLC; 0.1 ng/μL HRPL template; 0.3 mM dNTPs (0.075 mM each); 3% DMSO; 1.5 mM MgCl_2_; 0.01 mM MnCl_2 _and 0.05 U/μL Taq polymerase. For the Mutazyme library the concentrations of each reagent in 50 μL final volume were as follows: 370 nM RMLN; 370 nM RMLC, 40 ng/μL HRPL template; 0.8 mM dNTPs; 3% DMSO; and 0.05 U/μL Mutazyme DNA polymerase. Taq/MnCl_2 _and Mutazyme libraries were equimolarly mixed and transformed along with linearized vector (ratio equimolar library:vector, 8:1) into competent *S. cerevisiae *cells as described above.

### 3. High-throughput thermostability assay

Individual clones were picked and cultured in 96-well plates (Sero-well, Staffordshire, UK) containing 50 μL of minimal medium per well. In each plate, column number 6 was inoculated with standard (parental HRPL or VP), and one well (H1-control) was either not inoculated for HRPL libraries or inoculated with untransformed *S. cerevisiae *cells for VP-libraries. Plates were sealed to prevent evaporation and incubated at 30°C, 225 RPM and 80% relative humidity in a humidity shaker (Minitron-INFORS, Biogen, Spain). After 48 h, 160 μL of expression medium were added to each well, and the plates were incubated for 24 h. The plates (master plates) were centrifuged (Eppendorf 5810R centrifuge, Germany) for 5 min at 3000 × *g *at 4°C and 20 μL of supernatant was transferred from the master plate with the help of a robot (Liquid Handler Quadra 96-320, Tomtec, Hamden, CT, USA) onto the replica plate. Subsequently, 180 μL of stability buffer (10 mM sodium tartrate buffer pH 5.1 for VP-library and 10 mM Britton and Robinson buffer pH 6.0 for HRPL-library) were added to each replica and briefly stirred. Replica plate was duplicated with the help of the robot by transferring 50 μL of mixture to a thermocycler plate (Multiply PCR plate without skirt, neutral, Sarstedt, Germany) and 20 μL to the initial activity plate. Thermocycler plates were sealed with thermoresistant film (Deltalab, Spain) and incubated at the corresponding temperature using a thermocycler (MyCycler, Biorad, USA). Incubation took place for 10 min (so that the assessed activity was reduced 2/3 of the initial activity). Afterwards, thermocycler plates were placed on ice for 10 min and further incubated for 5 min at room temperature. 20 μL of supernatants were transferred from both thermocycler and initial activity plates to new plates to estimate the initial activities and residual activities values by adding ABTS containing specific buffers. For VP-libraries 180 μL of 100 mM sodium tartrate buffer pH 3.5 containing 2 mM ABTS and 0.1 mM H_2_O_2 _were added to each plate. For HRPL-libraries 180 μL of 100 mM sodium acetate buffer pH 5.0 containing 3 mM ABTS were added. Plates were stirred briefly and the absorption at 418 nm (ε_ABTS_^•+ ^= 36,000 M^-1 ^cm^-1^) was recorded in the plate reader (SPECTRAMax Plus 384, Molecular Devices, Sunnyvale, CA). The plates were incubated at room temperature until a green color developed, and the absorption was measured again. The same experiment was performed for both the initial activity plate and residual activity plate. Relative activities were calculated from the difference between the absorption after incubation and that of the initial measurement normalized against the parental type in the corresponding plate. Thermostability values came from the ratio between residual activities and initial activities values. To rule out false positives, two consecutive rescreenings were carried out according to the protocol previously reported [33] with some modifications. A third rescreening was incorporated to calculate the T_50 _of selected mutants.

#### First rescreening

aliquots of 5 μL of the best clones were removed from master plates to inoculate 50 μL of minimal media in new 96-well plates. Columns 1 and 12 (rows A and H) were not used to prevent the appearance of false positives. After 24 h of incubation at 30°C and 225 RPM, 5 μL were transferred to the adjacent wells and further incubated for 24 h. Finally, 160 μL of expression medium were added and plates were incubated for 24 h. Accordingly, every single mutant was grown in 4 wells. Parent types were subjected to the same procedure (lane D, wells 7-11). Plates were assessed using the same protocol of the screening described above but including not only an endpoint assay but also a kinetic assay. In the ABTS kinetic assay, linear absorption increases over a wide range of enzyme concentration (1-20 mU/mL) allowing the estimation of initial rates.

#### Second rescreening

an aliquot from the wells with the best clones of first rescreening was inoculated in 3 mL of YPD and incubated at 30°C and 225 RPM for 24 h. Plasmids from these cultures were extracted (Zymoprep yeast plasmid miniprep kit, Zymo Research). As the product of the zymoprep was very impure and the concentration of extracted DNA was very low, the shuttle vectors were transformed into super-competent *E. coli *cells (XL2-Blue, Stratagene) and plated onto LB-amp plates. Single colonies were picked and used to inoculate 5 mL LB-amp media and were grown overnight at 37°C and 225 RPM. Plasmids were then extracted (NucleoSpin^® ^Plasmid kit, Macherey-Nagel, Germany). *S. cerevisiae *was transformed with plasmids from the best mutants and also with parent type. Five colonies of every single mutant were picked and rescreened as described above (using both end-point and kinetic assays).

#### Third rescreening (T_50 _determination)

fresh transformants of selected mutants and parent types were cultivated (10 mL) in 100 mL flask for VP and HRPL production. Supernatants were subjected to a thermostability assay to accurate estimate their T_50 _using 96/384 well gradient thermocyclers (Mycycler, Biorad, US). Appropriate dilutions of supernatants were prepared with the help of the robot in such a way that aliquots of 20 μL give rise to a linear response in kinetic mode. 50 μL (from both selected mutants and parent types) were used for each point in the gradient scale. A temperature gradient profile ranging from 30 to 90°C was established. After 10 min of incubation, samples were removed and chilled out on ice for 10 min. Afterthat, samples of 20 μL were removed and incubated at room temperature for 5 min. Finally, samples were subjected to the same ABTS-based colorimetric assay described above for the screening. Thermostabilities values were deduced from the ratio between the residual activities incubated at different temperature points and the initial activity at room temperature.

### 4. Determination of thermostabilities in VP and HRPL parent types

Thermostabilities of 7H2-HRPL and 10C3-VP mutants were assessed mimicking the growth conditions established for the screening assay as described above. Two 96 well-plates containing 50 μL minimal media were inoculated with 7H2 and 10C3 respectively and cultivated until reaching functional expression following the conditions used for the assay. Afterwards, supernantants of 7H2 and 10C3 were pooled and employed to estimate their respective thermostabilities with the gradient thermocycler. The gradient of temperature was set at the following points (in°C): 30.0, 31.7, 34.8, 39.3, 45.3, 49.9, 53.0, 55.0, 56.8, 59.9, 64.3, 70.3, 75.0, 78.1 and 80 for the VP mutant and 35.0, 36.7, 39.8, 44.2, 50.2, 54.9, 58.0, 60.0, 61.1, 63.0, 65.6, 69.2, 72.1, 73.9, 75.0, 76.2, 78.0, 80.7, 84.3, 87.1, 89.0 and 90.0 for the HRPL mutant. The protocol followed the general rules described for the third re-screening.

### 5. DNA sequencing

Plasmid-containing variant HRPL and VP genes were sequenced by using a BigDye Terminator v 3.1 Cycle Sequencing Kit. Primers were designed with Fast-PCR software (University of Helsinki, Finland). Primers used for VP variants were: RMLN; 3R-direct (5'-GTTCCATCATCGCGTTCG-3'); 5F-reverse (5'-GGATTCCTTTCTTCTTGG-3') and RMLC. For HRPL primers were: RMLN; PM1FS (5'-ACGACTTCCAGGTCCCTGACCAAGC-3'); PM1RS (5'-TCAATGTCCGCGTTCGCAGGGA-3') and RMLC.

### 6. Protein modelling

We carried out a search in the Protein Data Bank for proteins with known structural homology to laccase PM1. The most similar protein to PM1 was a laccase from *Trametes trogii *(crystal structure solved with a resolution of 1.58 Å), showing 97% sequence identity (PDB id: 2hrgA) [34]. A model from the Swiss-Model protein automated modelling server was generated http://swissmodel.expasy.org/ and analyzed with DeepView/Swiss-Pdb Viewer.

## Results and discussion

### 1. Library construction

**Figure 1:**
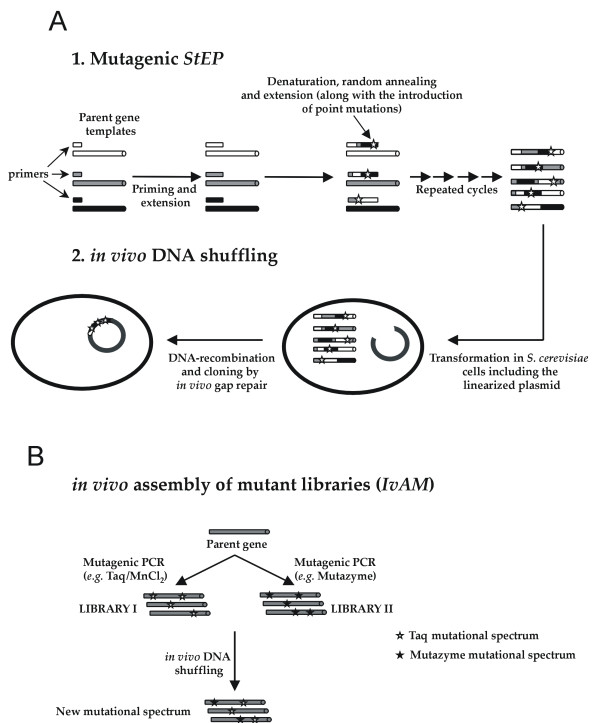
**schematic representation of methods employed for the construction of VP (A) and HRPL (B) mutant libraries**.

VP and HRPL variants come from laboratory evolution approaches to be functionally expressed in *Saccharomyces cerevisiae *([29] and unpublished material). Therefore, the array of variants used as starting points for enhancing thermostability, in principle only were going to harbor mutations which conferred higher secretion levels and/or better kinetics. Both VP and HRPL native signal peptides were replaced by the α-factor pre-proleader to promote secretion in yeast [35]. Thus, the whole fusion proteins (α-VP and α-HRPL) were subjected to directed evolution in order to enhance their functional expression; *i.e*. the accumulation of neutral and beneficial mutations took place both in the α-factor pre-proleader as well as in mature protein. If high fidelity DNA recombination strategies are used, where only accumulated mutations recombine each other based on the number of crossovers generated in the protocol, it is highly likely that the discovery of thermostable variants become a complex task, since all these mutations have been unmasked from improvements in total activities; *i.e*. the sum of secretion levels plus the specific activity. Hence, we decided to tackle the library design by combining the already accumulated mutations with the generation of new ones randomly incorporated in the frame of mutagenic DNA recombination fashion approaches (Fig. 1). For the VP library the well known *Staggered Extension Process *(*StEP*) [30] was slightly modified by decreasing templates concentration with the aim of provoking amplification mistakes by the Taq DNA-Polymerase which has a high intrinsic error rate because of the lack of 3'-5'proofreading exonuclease activity. The mutational rate was adjusted to 1-2 mutations per whole gene [36]. Afterwards, and instead of *in vitro *ligating the *StEP *products with the linearized plasmid to give rise to individual autonomously replicating vectors as usually proceeds, we thought that might be interesting to further recombine the pool of mutagenized/crossover containing genes in an *in vivo *approach with the aim of expanding the library diversity. Thus, *StEP *products were further subjected to an *in vivo *DNA-shuffling process [31,37,38] taking advantage of the high level of homologous recombination of *S. cerevisiae *apparatus as described in the Material & Methods section. With this strategy, new crossover events were generated that otherwise hardly could be achieved by using conventional *in vitro *or *in vivo *recombination methods independently. In the case of HRPL library, the only starting template gene was subjected to the *in vivo *assembly of mutant libraries with different mutational spectrum (*IvAM*) [32], to guarantee the enrichment of the library in different mutational types and bias. With this simple approach, the homologous recombination, reparation and *in vivo *cloning of mutant genes along with their *in vivo *DNA-shuffling, in one single step were achieved.

### 2. High-throughput screening assay

**Figure 2:**
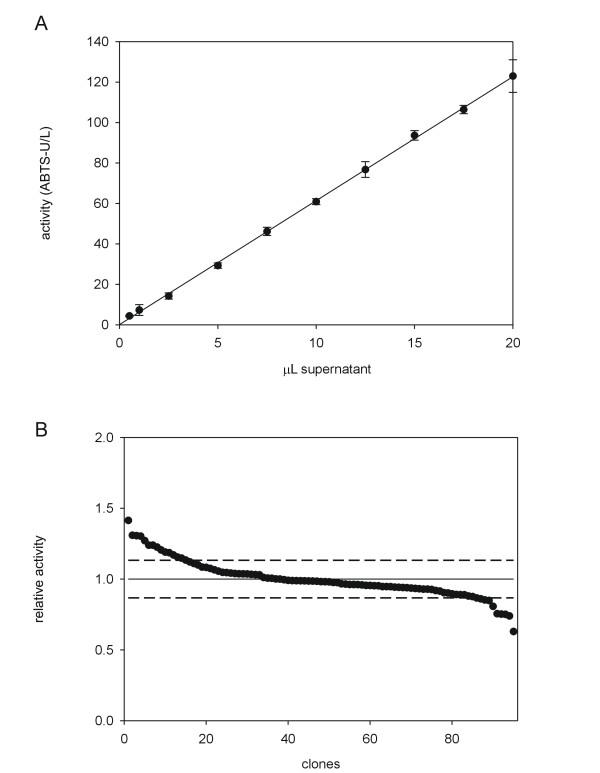
**validation of the colorimetric assay for the VP-library**. A) Linearity of the assay. Each point represents the average of 8 experiments (8 wells). B) Activities of VP-10C3 plotted in descending order. Dashed lines indicate the variation coefficient of the assay. *S. cerevisiae *cells were transformed with pJRoC30-VP-10C3 and plated on SC dropout plates. Individual colonies were picked and inoculated in a 96 well-plate. The activities of the clones were evaluated from fresh supernatant preparations.

ABTS was chosen as substrate for the screening assay since it has a reliable response, high sensitivity and hardly interferes with the main components of culture broth. Indeed, in our former works ABTS was validated in the frame of directed laccase evolution for several purposes [33,38-40]. However, the response of this substrate against the secreted VP mutants by *S. cerevisiae *was unknown and therefore we had to validate it before starting the thermostability studies. First, the relationship between absorbance and VP concentration was evaluated. Fresh transformants containing VP-10C3 mutant (the best parent type) were inoculated in a 96-well plate, VP-10C3 was expressed and different volumes of supernatant were assessed. A linear behavior between the amount of VP and the response of the assay was found (Fig. 2A). The coefficient of variation for the ABTS in kinetic mode was 13%, which is an acceptable value to study mutagenic libraries engineered for directed evolution experiments [9] (Fig. 2B).

**Figure 3:**
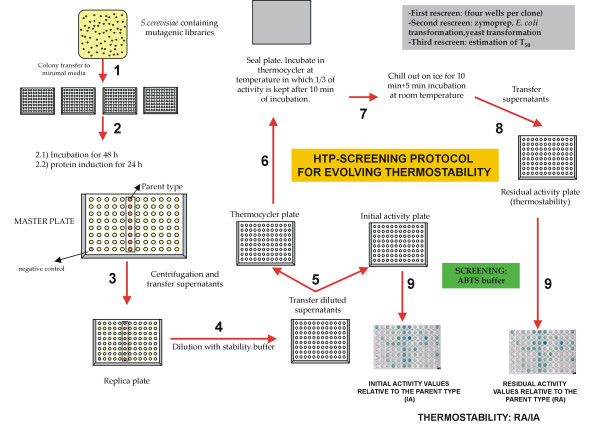
**high-throughput protocol used for screening thermostability in VP- and HRPL-libraries**. Further details in Material & Methods Section.

For the thermostability assay, we first had to set the temperature under which the mutant libraries would be stressed. Accordingly, the two best parent types of both VP and HRPL libraries were tested in order to know their respective thermostabilities. Taking into account that micro-fermentation conditions (*i.e*. in 96 well plates) were far away from an ideal large-scale fermentation in terms of oxygen availability, surface stirring and length of incubation time, VP-10C3 and HRPL-7H2 mutants were produced under such a limited growth conditions and evaluated to obtain the closest real value of thermostability in the screening assay (further details in Material & Methods section). In principle, an appropriate enzyme heat treatment for the screening is generally chosen so that the residual activity is about one-third of the initial activity [9]. At 60°C and 68°C, VP-10C3 and HRPL-7H2 mutants kept *c.a*. 30% of their initial ABTS-activity values, and those temperatures were selected for the screening. At this point, it is important to highlight that both VP and HRPL libraries were functionally secreted by yeast, which made the development of the assay faster, reliable and simple since it was not necessary to include cell lysis steps, thus avoiding possible interferences with the complex lysate mixture. Besides, both VP and HRPL secretion levels were previously improved by several iterative rounds of directed evolution to provide: i) short incubations times for functional secretion (24 h) after protein induction that becomes essential for the episomal plasmid stability; and ii) a high level of activity in supernatants (getting measurements from kinetic mode in a few seconds with turnover rates ranging from 0.5 to 1 ABTS-Units/mL). Taking advantage of the high level of activity in the culture broth, supernatants of respective libraries were previously diluted in suitable corresponding stability buffers to correctly assess initial and residual activities (Fig. 3). The final values of thermostability came from the ratio of residual activity to initial activity (RA/IA) normalized with the corresponding parent type. The initial activity value reflected the total activity of the mutant and was highly useful to prevent the selection of mutants with improved stability by greatly reduced activity.

### 3. Library analysis

**Figure 4:**
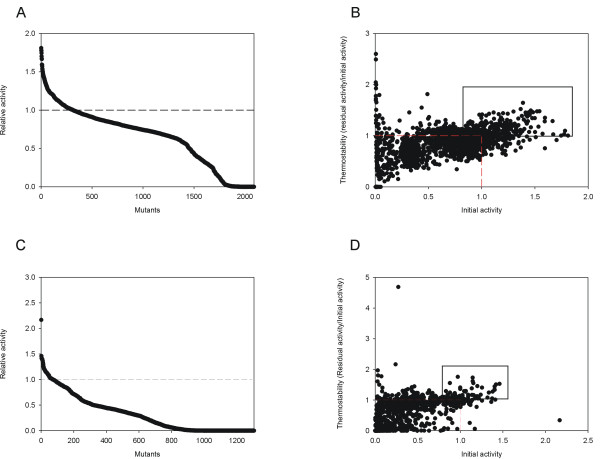
**directed evolution landscapes**. A) Activities of clones from the library of VP mutants prepared by mutagenic *StEP *+ *in vivo *DNA shuffling, plotted in descending order. B) Initial activities *vs *thermostabilities in VP mutants. Red dashed lines represent VP-10C3 parent type. Mutants selected for further re-screens are squared (see Material & Methods section for details). C) Activities of clones from the library of HRPL mutants prepared by *IvAM*, plotted in descending order. D) Initial activities *vs *thermostabilities in HRPL mutants. Red dashed lines represent 7H2 parent type. Mutants selected for further re-screens are squared.

Under the above premises, two libraries of ~2000 clones were independently constructed and explored for VP and HRPL (Fig. 4). The estimated coefficients of variance for the landscapes of initial activity and residual activity were below 12%. Three consecutive re-screens were incorporated to ensure the selection of mutant *hits *in protein function. Systematically, the best 50 clones retaining ~0.7 fold the activity of parent type and showing improvements in thermostability were selected and double rescreened from supernatants and new fresh transformants. Finally, best mutants were subjected to a third rescreen to estimate their T_50_; *i.e*. the transition midpoint of the inactivation curve of the protein as a function of temperature, which in our case was defined as the temperature at which the enzyme loses 50% of its activity following incubation for 10 min.

**Figure 5:**
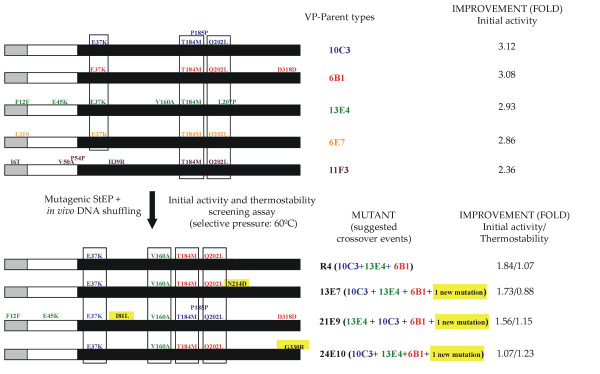
**improved variants screened from VP-library**. The α factor pre-leader is represented in grey, the α factor pro-leader in white and the mature protein in black. Suggested recombination events are indicated in different colours. New point mutations are highlighted in yellow.

**Figure 6:**
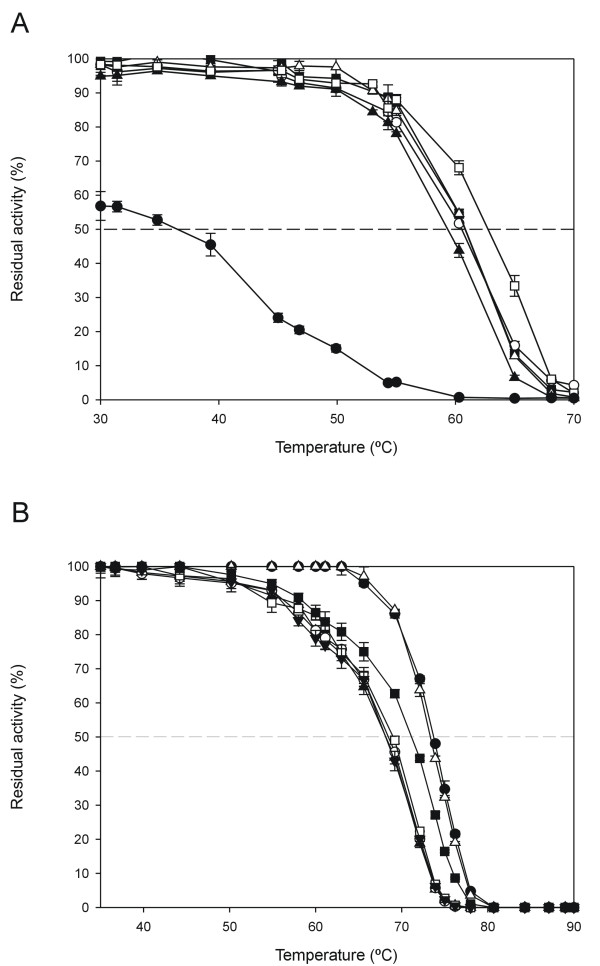
**A) T_50 _for VP parent types and different mutants of the evolutionary process**. White squares, 24E10 mutant; black triangles, R4 mutant; black squares, 10C3 mutant (best parent type from 3^rd ^generation); white circles, best parent type from 2^nd ^generation; white triangles, best parent type from 1^st ^generation; black circles, native VP expressed in *E. coli *after *in vitro *refolded from inclusion bodies. B) T_50 _for HRPL parent types and mutants of the *in vitro *evolution. Black circle, 1D11 mutant (4^th ^generation); white triangle, 11A2 mutant (4^th ^generation); black triangle, 7H2 mutant (5^th ^generation); white circle, 6C8 mutant (6^th ^generation); white square, 5H12 mutant (6^th ^generation); black down triangle, 10B1 mutant (6^th ^generation); black square, 16B10 mutant (6^th ^generation). Each point, including the standard deviation, comes from three independent experiments.

The best four mutants of VP library, with significant improvements in their activities and/or thermostabilities, were sequenced (Fig. 5). These variants shared the common feature of incorporating 4 mutations (E37K, V160A, T184M and Q202L) accumulated round after round of evolution in the same mature protein. Interestingly, the best variant of activity (R4 mutant with 3180 ± 30 ABTS-Units/L) only contained the mentioned 4 mutations after 4 cycles of evolution. The remaining selected variants displayed a negative epistatic effect, *i.e*. the general combination of mutations is beneficial but at least one individual mutation is not, after incorporating new point mutations (with improvement ranging from 1.7 to 1.1 fold *vs *best parent type, 10C3 mutant). It is noteworthy that the best thermostable variant (24E10 mutant, with 1.23-fold better stability than 10C3) contains the same four mutations as R4 plus mutation G330R, which logically is responsible for the improvement in the thermostability. Mutation G330R shifted the T_50 _2.2°C, from 60.5°C to 62.7°C (Fig. 6A), but decreased the activity of the variant almost 0.77 fold. Indeed, the improvement in protein stability came at the cost of reducing activity (the well known tradeoff that usually appears between activity and stability for many single point mutations). However, the recombination method designed for this experiment (mutagenic *StEP *+ *in vivo *DNA shuffling) made possible to join the four beneficial mutations for the activity which somehow buffered the effect of incorporating the stabilizing mutation G330R, giving rise to an enzyme with similar activity to the parent types but more thermostable. Mutation G330R is placed at the C-terminal tail of VP, a controversial region that, although is not involved in VP oxidation of Mn^2+ ^as initially suggested for the C-tail of *Phanerochaete chrysosporium *MnP [41], presents a high mobility that prevented to fix the position of the last 12 residues when the VP crystal structure was solved (PDB entry 2BOQ). This highly-mobile region could contribute to the thermal denaturation of the protein, and it is possible to speculate that the introduction of an arginine side-chain at position-330 could stabilize the region, and the whole VP protein, by establishing some new interaction/s, whose nature cannot be determined due to the lack of valuable crystallographic data for the C-terminal tail of VP.

**Figure 7:**
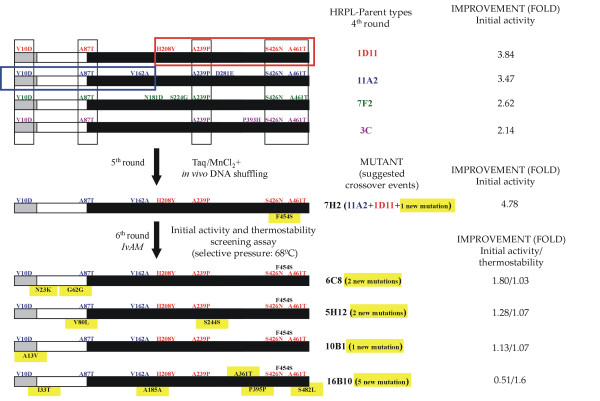
**improved variants screened from HRPL-library**. α-factor pre-leader is represented in grey, α-factor proleader in white and mature protein in black. Suggested recombination events are indicated in different colours. New point mutations are highlighted in yellow. Squared in red and blue is highlighted the recombination event that took place between 11D11 and 11A2 to generate 7H2 mutant in the 4^th ^cycle of evolution.

**Figure 8:**
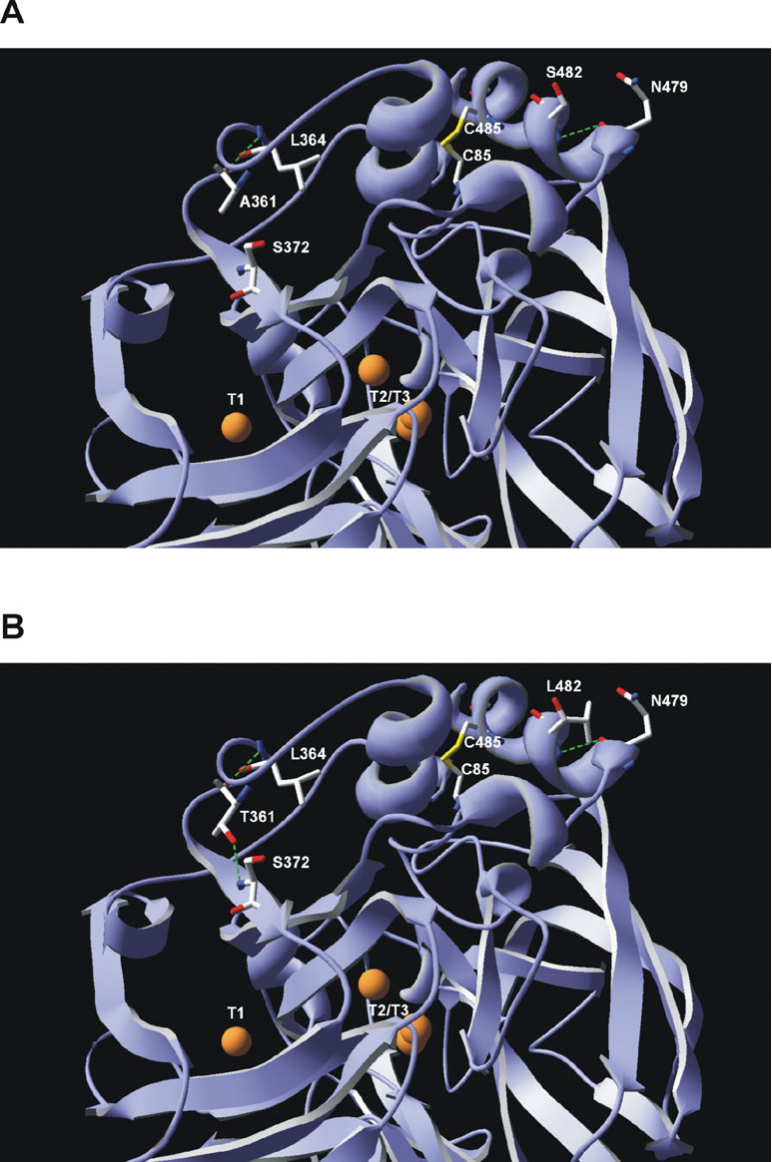
**location and surroundings of stabilizing mutations in HRPL**. A) parent type. B) 16B10 variant. The orange spheres represent Cu atoms.

HRPL-library was generated by *IvAM *from 7H2 mutant, the best variant of the 5^th ^round of evolution for total activity enhancement (Fig. 7). 7H2 was a good candidate to improve the thermostability: during the former round of evolution by mutagenic *in vivo *DNA-shuffling, although its activity was improved almost 5-fold (achieving 1000 ± 24 ABTS-Units/L), its stability diminished considerably, with a decrease in the T_50 _of ~5°C *vs *1D11 and 11A2 parent types (Fig. 6B). As main consequence of the drop in the T_50_, 7H2 was unstable during long-term storage (loosing about 30% of its activity after 15 days at 4°C). In the context of directed evolution experiments, this effect is not surprising and there are many examples in literature about falls in stability because of introducing beneficial but destabilizing mutations for enhanced activity [3]. Taking into account that 7H2 was created from a single crossover event between 1D11 and 11A2 variants (Fig. 7), and that both parent types shares similar T_50 _values (73.7 and 73.3°C respectively, Fig. 6B) it became clear that the only new mutation incorporated in 7H2 (F454S) was responsible for the dramatic drop in the T_50_. The mutation was mapped in a model based on the *Trametes trogii *laccase (97% identity) crystal structure [34]. Phe454 is placed in a region close to the T1 cooper site, the place where the reducing substrate binds. The change from Phe to Ser at this position seems to disrupt several interactions with neighboring residues, which probably affects stability. Best mutants of the thermostability cycle where sequenced (Table 1) and in all cases new mutations at the pre- or pro-leader were introduced benefiting the secretion levels by yeast (with improvement in total activities ranging from 1800 to 500 ABTS-Units/L). The best thermostable mutant (16B10 variant) displayed 1.6-fold better stability than 7H2, shifting its T_50 _value over 3°C, but at the cost of reducing its activity by half (Fig. 6B and Fig. 7). In spite of the fact that *IvAM *method takes advantage of mixing different mutational profiles created by polymerases with broad differences in mutational bias (Table 1), the constraint of starting from only one single parent type hindered to find appropriate crossovers events (as happened in VP-libraries) that otherwise could have helped to prevent the detrimental effect on activity of introducing stabilizing mutations. 16B10 harbours 4 mutations in mature protein (two synonymous). Mutations A361T and S482L are placed at the surface of the protein, close to the C-end. Both mutations are at least 21 Å from the nearest catalytic copper atom (Table 1, Fig. 8). Ala361 is located in a loop and interacts with Leu364 by a hydrogen bond. The change from Ala to Thr at position 361, kept the mentioned interaction and allowed one additional hydrogen bond with Ser372 placed in a beta-sheet. This structural reinforcement upon mutation might confer rigidity to the protein enhancing its stability. Mutation S482L is in the neighbourhood of the disulfide bridge between Cys485 and Cys85. Ser482 is part of a α-helix and establishes a hydrogen bond with Gln479 of same motif. Inspection of the protein model suggests that the S482L mutation does not interrupt such a bond. The change of a polar amino acid by a bigger hydrophobic one might allow establishing hydrophobic interactions with surrounding residues which may further stabilize the protein structure at this region.

**Table 1:** HRPL selected mutants generated by *IvAM*

Mutant	Amino acid Substitution	Nucleotide change	Mutation type	Location	Secondary structure motif	Distance to the T1 Site (Å)	Distance to the T2/T3 (Å)
6C8	N23K	AA**C**69AA**A**	Tv	Pro-leader			
	G62G	_6_GG**G**186GG**A**_11_	Ts	Pro-leader			
			
5H12	V80L	**G**TA238**C**TA	Tv	Pro-leader			
	S244S	_8_TC**G**1005TC**C**_14_	Tv	Mature protein			
			
10B1	A13V	G**C**A38G**T**A	Ts	Pre-leader			

	I33T	A**T**T98A**C**T	Ts	Pro-leader			
	A185A	_21_GC**T**828GC**C**_13_	Ts	Mature protein			
16B10	A361T	**G**CG1354**A**CG	Ts	Mature protein	loop	23	21
	P395P	_7_CC**C**1458CC**T**_13_	Ts	Mature protein			
	S482L	T**C**G1718T**T**G	Ts	Mature protein	sheet	34	24

Synonymous mutations are underlined; nucleotide change is highlighted in bold; subscript numbers on codons indicate codon usage. Tv, transversions; Ts, transitions.

## Conclusions

In summary, *S. cerevisiae *is a valuable cell factory for the directed evolution of ligninolytic enzymes for thermostability and taking together, the VP and the HRPL evolved variants share several common features. First, the thermostability improvements obtained for both VP and HRPL systems are especially significant near the enzyme inactivation temperatures: the best VP (24E10) showed ~30% of its maximal activity at 65°C (3-fold more than the initial VP) and the best laccase (16B10) up to 40% of its maximal activity at 72°C (over 10-fold more than the corresponding parent type). Second, an apparently inherent tradeoff between activity and stability appeared in both enzymes for different amino acid substitutions. Although not physically incompatible, in general protein scaffolds activity and thermostability tend to act as communicating vessels and the laboratory design of any of them usually come at the cost of its counterpart. For protein engineers, to find single mutations which improve both properties simultaneously is extremely difficult. In nature, stability is under selection just in the case that it is required for biochemical function, hence mutations which join activity and stability are rare taking into account the genetic drift and that a selective pressure towards both features at the same time is not frequently exerted. It has been reported that in principle is easier to evolve thermostability while keeping activity than vice versa, although recent research indicates that evolving activity while maintaining stability can be accomplished as well [1,39]. We have demonstrated that the generation of complex crossover events along with the introduction of new mutations facilitates the improvement in the stability of ligninolytic oxidoreductases buffering the drops on their activities. In the evolutionary scenario, the recombination methods described in this work for the generation of diversity along with the screening assay engineered for this specific task can be valuable tools not only to tailor thermostable ligninolytic oxidoreductases but also other enzymatic systems.

## Abbreviations

ABTS: 2,2'-azino-bis (3 ethylbenzothiazoline 6 sulfonic acid); DMSO: dimethylsulfoxide; HRPL: High Redox Potential Laccases; IvAM: *In vivo *Assembly of Mutant libraries with different mutational spectra; RA/IA: Residual Activity/Initial Activity; StEP: Staggered Extension Process; T_50_: Temperature at which the enzyme loses 50% of its activity following incubation for 10 minutes; VP: Versatile Peroxidase.

## Competing interests

The authors declare that they have no competing interests.

## Authors' contributions

The PhD students EGR and DM carried out all the experiments on VP and HRPL respectively under the supervision of MA. ATM provided background on VP and co-supervised EGR. MA wrote the first draft which was revised by ATM and AB. MA coordinated the final version of the paper, which was read and approved by all authors.

## References

[B1] Bloom JD Labthavikul ST Otey CR Arnold FH Protein stability promotes evolvability Proc Natl Acad Sci USA 2006 103 5869 5874 10.1073/pnas.0510098103 16581913 PMC1458665

[B2] Bloom JD Wilke CO Arnold FH Adami C Stability and the evolvability of function in a model protein Biophys J 2004 86 2758 2764 10.1016/S0006-3495(04)74329-5 15111394 PMC1304146

[B3] Bloom JD Arnold FH In the light of directed evolution: Pathways of adaptive protein evolution Proc Natl Acad Sci USA 2009 106 9995 10000 10.1073/pnas.0901522106 19528653 PMC2702793

[B4] Watanabe K Ohkuri T Yokobori S Yamagishi A Designing thermostable proteins: Ancestral mutants of 3-isopropylmalate dehydrogenase designed by using a phylogenetic tree J Mol Biol 2006 355 664 674 10.1016/j.jmb.2005.10.011 16309701

[B5] Ogino H Ishikawa H enzymes which are stable in the presence of organic solvents J Biosci Bioeng 2001 91 109 116 10.1263/jbb.91.109 16232960

[B6] Arnold FH Engineering enzymes for nonaqueous solvents Trends Biotechnol 1990 8 244 249 10.1016/0167-7799(90)90186-2 1366732

[B7] Suen WC Zhang NY Xiao L Madison V Zaks A Improved activity and thermostability of *Candida antarctica *lipase B by DNA family shuffling Protein Eng 2004 17 133 140 10.1093/protein/gzh017 15047909

[B8] Johannes TW Woodyer RD Zhao HM Directed evolution of a thermostable phosphite dehydrogenase for NAD(P)H regeneration Appl Environ Microb 2005 71 5728 5734 10.1128/AEM.71.10.5728-5734.2005 PMC1265921 16204481

[B9] Arnold FH Georgiou G Directed Enzyme Evolution: screening and selection methods Methods in Molecular Biology 2003 231 Humana Press, Totowa, New Jersey12824608

[B10] Reetz MT Carballeira JD Vogel A Iterative saturation mutagenesis on the basis of B factors as a strategy for increasing protein thermostability Angew Chem Int Ed 2006 45 7745 7751 10.1002/anie.200602795 17075931

[B11] Bommarius AS Broering JM Chaparro-Riggers JF Polizzi KM High-throughput screening for enhanced protein stability Curr Opin Biotech 2006 17 606 610 10.1016/j.copbio.2006.10.001 17049838

[B12] López Camacho C Salgado J Lequerica JL Madarro A Ballestar E Franco L Polaina J Amino acid substitution enhancing thermostability of *Bacillus polymixa *beta-glucosidase A Biochem J 1996 314 833 838 8615777 10.1042/bj3140833 PMC1217132

[B13] Miyazaki K Takenouchi M Kondo H Noro N Suzuki M Tsuda S Thermal stabilization of *Bacillus subtilis *family-11 xylanase by directed evolution J Biol Chem 2006 281 10236 10242 10.1074/jbc.M511948200 16467302

[B14] Giver L Gershenson A Freskgard PO Arnold FH Directed evolution of a thermostable esterase Proc Natl Acad Sci USA 1998 95 12809 12813 10.1073/pnas.95.22.12809 9788996 PMC23604

[B15] Arnold FH Giver L Gershenson A Zhao HM Miyazaki K Directed evolution of mesophilic enzymes into their thermophilic counterparts Proc Natl Acad Sci USA 1999 870 400 403 10.1111/j.1749-6632.1999.tb08913.x 10415508

[B16] Hao J Berry A A thermostable variant of fructose bisphosphate aldolase constructed by directed evolution also shows increased stability in organic solvents Protein Eng 2004 17 689 697 10.1093/protein/gzh081 15531627

[B17] Morawski B Quan S Arnold FH Functional expression and stabilization of horseradish peroxidase by directed evolution in *Saccharomyces cerevisiae* Biotechnol Bioeng 2001 76 99 107 10.1002/bit.1149 11505379

[B18] Salazar O Cirino PC Arnold FH Thermostabilization of a cytochrome P450 peroxygenase Chem Bio Chem 2003 4 891 893 12964165 10.1002/cbic.200300660

[B19] Reading NS Aust SD Engineering a disulfide bond in recombinant manganase peroxidase results in increased thermostability Biotechnol Progr 2000 16 326 333 10.1021/bp0000151 10835231

[B20] Sun LH Petrounia IP Yagasaki M Bandara G Arnold FH Expression and stabilization of galactose oxidase in *Escherichia coli *by directed evolution Protein Eng 2001 14 699 704 10.1093/protein/14.9.699 11707617

[B21] Kunamneni A Camarero S García-Burgos C Plou FJ Ballesteros A Alcalde M Engineering and Applications of fungal laccases for organic synthesis Microb Cell Fact 2008 7 10.1186/1475-2859-7-32 19019256 PMC2613868

[B22] Alcalde M Ferrer M Plou FJ Ballesteros A Environmental biocatalysis: from remediation with enzymes to novel green processes Trends Biotechnol 2006 24 281 287 10.1016/j.tibtech.2006.04.002 16647150

[B23] Martínez AT Ruiz-Dueñas FJ Martínez MJ del Río JC Gutiérrez A Enzymatic delignification of plant cell wall: from nature to mill Curr Opin Biotechnol 2009 20 348 357 10.1016/j.copbio.2009.05.002 19502047

[B24] Alcalde M Polaina J, MacCabe AP Laccase: biological functions, molecular structure and industrial applications Industrial Enzymes: structure, functions and applications 2007 New York, Springer 459 474

[B25] Riva S Laccases: blue enzymes for green chemistry Trends Biotechnol 2006 24 219 226 10.1016/j.tibtech.2006.03.006 16574262

[B26] Ruiz-Dueñas FJ Morales M García E Miki Y Martínez MJ Martínez AT Substrate oxidation sites in versatile peroxidase and other basidiomycete peroxidases J Exp Bot 2009 60 441 452 10.1093/jxb/ern261 18987391

[B27] Ruiz-Duenas FJ Martinez MJ Martinez AT Heterologous expression of *Pleurotus eryngii *peroxidase confirms its ability to oxidize Mn^2+ ^and different aromatic substrates Appl Environ Microbiol 1999 65 4705 4707 10508113 10.1128/aem.65.10.4705-4707.1999 PMC91631

[B28] Coll PM Tabernero C Santamaria R Perez P Characterization and structural analysis of the laccase I gene from the newly isolated ligninolytic basidiomycete PM1 (CECT 2971) Appl Environ Microbiol 1993 59 4129 4135 8285710 10.1128/aem.59.12.4129-4135.1993 PMC195876

[B29] García E Martínez MJ Dueñas J Martínez AT Alcalde M High redox potential peroxidases engineered by directed evolution Patent n° 200930157. Spain 2009

[B30] Zhao HM Giver L Shao ZX Affholter JA Arnold FH Molecular evolution by staggered extension process (StEP) *in vitro *recombination Nature Biotech 1998 16 258 261 10.1038/nbt0398-258 9528005

[B31] Okkels JS Svendsen A *In vivo *gene shuffling in yeast: a fast and easy method for directed evolution of enzymes Enzyme Functionality: Design, Engineering and Screening 2004 Marcel Dekker, Inc. New York 413 424

[B32] Zumárraga M Camarero S Shleev S Martínez-Arias A Ballesteros A Plou FJ Alcalde M Altering the laccase functionality by *in vivo *assembly of mutant libraries with different mutational spectra Proteins 2008 71 250 260 10.1002/prot.21699 17932916

[B33] Zumárraga M Domínguez CV Camarero S Shleev S Polaina J Martínez-Arias A Ferrer ML de Lacey A Fernández V Ballesteros A Plou FJ Alcalde M Combinatorial Saturation Mutagenesis of the Myceliophthora thermophila laccase T2 mutant: the connection between the C-terminal plug and the conserved 509VSG511 tripeptide Comb Chem High T Scr 2008 11 807 816 10.2174/138620708786734235 19075602

[B34] Matera I Gullotto A Tilli S Ferraroni M Scozzafava A Briganti F Crystal structure of the blue multicopper oxidase from the white-rot fungus *Trametes trogii *complexed with *p*-toluate Inorg Chim Acta 2008 361 4129 4137 10.1016/j.ica.2008.03.091

[B35] Shuster JR Gene expression in yeast: protein secretion Curr Opin Biotechnol 1991 2 685 690 10.1016/0958-1669(91)90035-4 1367718

[B36] Tracewell CA Arnold FH Directed ezyme evolution: climbing fitness peaks one amino acid at a time Curr Opin Chem Biol 2009 13 3 9 10.1016/j.cbpa.2009.01.017 19249235 PMC2703427

[B37] Cherry JR Lamsa MH Schneider P Vind J Svendsen A Jones A Pedersen AH Directed Evolution of a fungal peroxidase Nature Biotech 1999 17 379 384 10.1038/7939 10207888

[B38] Bulter T Alcalde M Sieber V Meinhold P Schlachtbauer C Arnold FH Functional expression of a fungal laccase in *Saccharomyces cerevisiae *by directed evolution Appl Environ Microb 2003 69 987 995 10.1128/AEM.69.2.987-995.2003 PMC143632 12571021

[B39] Zumárraga M Bulter T Shleev S Polaina J Martínez-Arias A Plou FJ Ballesteros A Alcalde M *In vitro *evolution of a fungal laccase in high concentrations of organic cosolvents Chem Biol 2007 14 1052 1064 10.1016/j.chembiol.2007.08.010 17884637

[B40] Alcalde M Zumárraga M Polaina J Ballesteros A Plou FJ Combinatorial saturation mutagenesis by *in vivo *overlap extension for the engineering of fungal laccases Comb Chem High T Scr 2006 9 719 727 10.2174/138620706779026079 17168677

[B41] Ruiz-Dueñas FJ Morales M Pérez-Boada M Choinowski T Martínez MJ Piontek K Martínez AT Manganese oxidation site in *Pleurotus eryngii *versatile peroxidase: A site-directed mutagenesis, kinetic, and crystallographic study Biochemistry 2007 46 66 77 10.1021/bi061542h 17198376

